# Rodrigo Corrêa de Oliveira (★1956 †2023)

**DOI:** 10.1590/0037-8682-0547-2023

**Published:** 2023-12-08

**Authors:** Juliana de Assis Silva Gomes, Olindo Assis Martins-Filho

**Affiliations:** 1 Universidade Federal de Minas Gerais, Instituto de Ciências Biológicas, Departamento de Morfologia, Laboratório de Biologia das Interações Celulares, Belo Horizonte, MG, Brasil.; 2 Grupo Integrado de Pesquisa em Biomarcadores, Instituto René Rachou, FIOCRUZ, Belo Horizonte, MG, Brasil.

Rodrigo Corrêa de Oliveira, a distinguished researcher in the immunology field, was born on April 6, 1957 in Belo Horizonte, Minas Gerais. He was the son of Antonio Correa Oliveira and Cecília Coelho Correia de Oliveira and married to Andrea Gazzinelli Correa de Oliveira, with whom he had two daughters, Ana Carolina and Juliana. His family has always been a source of immense pride and joy to him. 

**Figure:**
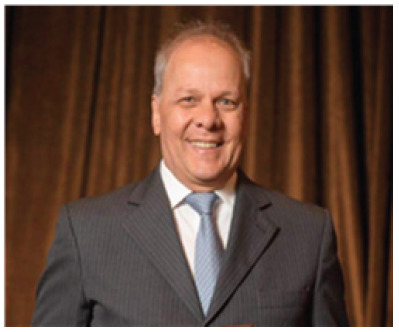

Rodrigo passed away on October 27, 2023 at 66 years of age. His life was marked by his commitment and love for research, leaving an indelible example for many scientists and a legacy of dedication and innovation. His journey towards scientific excellence began early when he exhibited curiosity and passion for understanding of the immunological mechanisms associated with infectious diseases. 

Rodrigo's academic journey was fulfilled by unlimited dreams. He received his bachelor’s degree in biology from the Universidade Federal de Minas Gerais (UFMG), MG, Brazil, in 1980^1^. At the beginning of his academic training, he became interested in immunology and worked as an undergraduate fellow at the Parasite Immunology Laboratory under the supervision of Professor Tomaz Aroldo da Mota Santos. In 1980, he published the first study on *Schistosoma mansoni* infection in a murine model. In 1981, he obtained his master’s degree from the Department of Biochemistry and Immunology, Instituto de Ciências Biológicas (ICB), UFMG, MG, Brazil, under the supervision of Professor Tomaz Aroldo da Mota Santos^1^. He then obtained his Ph.D. degree in immunology at the prestigious Johns Hopkins University in Baltimore, MD, USA, under the supervision of Dr. Stefanie James and Dr. Alan Sher (1982-1985)^1^. In 1991, he worked as a post-doctoral fellow at the DNAX Research Institute in Palo Alto, CA, USA, with Dr. Robert Coffman, who conducted the first studies on the effect of cytokines on the differentiation of type 1 and 2 helper T-cells^2^.

His groundbreaking research in immunology, particularly in the field of parasitic diseases, earned him international recognition and respect from peers and colleagues. Dr Correa-Oliveira started his researcher career at the Fundação Ezequiel Dias, FUNED, MG, Brazil in 1985 and then became a Full Researcher at the Instituto René Rachou (IRR) FIOCRUZ- Minas Gerais in 1986, where he was the Head of the Cellular and Molecular Immunology Laboratory and became Director in 2009 until his retirement in 2012. He was elected President of the Brazilian Society of Immunology in 2001. He acted as vice-president of the Tropical Disease Research (TDR) Board of the World Health Organization (WHO) (2006-2007) and the Chairperson of the Pathogenesis Committee, representing Brazil at the TDR-WHO Board and at the Pan American Health Organization (PAHO). He was also a member of the Research Council at Conselho Nacional de Desenvolvimento Científico e Tecnológico (CNPq), Fundação de Amparo à Pesquisa do Estado de Minas Gerais (FAPEMIG), and of the European Community.

Since 2011, he was a collaborating professor at the Nursing School of the Universidade Federal de Minas Gerais, visiting professor at the Universidade Federal de Ouro Preto, adjunct professor at the George Washington University School of Medicine and Health Sciences, and a member of the Scientific Advisory Board of the Institute of Tropical Medicine of University of Nova de Lisboa. Dr Rodrigo also mentored the implementation of the Immunology Research Laboratory at the University of Vale do Rio Doce, UNIVALE in Governador Valadares, Minas Gerais, Brazil. He was nominated as a full member of the Brazilian Academy of Sciences, the World Academy of Sciences for the Advancement of Science in Developing Countries, and International Fellow of American Society of Tropical Medicine and Hygiene^3^. 

In 2017, Dr. Rodrigo was nominated as the vice-president of the Research and Biological Collections at the Fundação Oswaldo Cruz (2017-2023). Thereafter, he was referred to as the Special Advisor of the FIOCRUZ Presidency, dedicating his work to the establishment of partnerships between the FIOCRUZ and international institutions.

Throughout his academic and scientific career, Dr. Rodrigo has been honored for several titles and prizes. Notably, he was designated as an outstanding alumnus of the UFMG and received the Honor for Health Merit from the State of Minas Gerais, the Carlos Chagas Medal, and the Medal of the Latin American Society of Pathology. He was a Research Fellow 1A at the CNPq, published 361 articles in high-impact journals and eight book chapters, and shared the authorship of three patents^1^. He has been cited 14,974 times (h-index = 71), occupying the 6^th^ position in the Brazilian ranking of immunologists^4^.

Dr. Rodrigo contributed to the training of students, professors, and researchers worldwide, supervising 25 Master’s students, 46 PhD students, and 27 post-doctoral fellows^1^. His research has focused on the analysis of immune responses to infectious and parasitic diseases, including *Schistosoma mansoni, Trypanosoma cruzi*, *Mycobacterium leprae*, geohelminths, and, more recently, the Zika and dengue viruses. His research aims to understand the roles of different immune mechanisms in the development of pathology and resistance to infection. Studies conducted under Rodrigo’s leadership have contributed significantly to the elucidation of the immunological pathways underlying infectious/parasitic infections in animal models and humans. His brilliant mind supported the continuous growth of his outstanding career, and positively impacted the lives of countless scientists who inevitably became close friends.

It is worth remembering that, in addition to his love for research and family, Rodrigo was also fascinated with soccer, especially with the Clube Atlético Mineiro, and was a dedicated road traveler of the Minas Gerais mountains and landscapes. Rodrigo was charismatic, friendly, generous, creative, and willing to help. He did not know how to say no to anyone who approached him. He loved talking about science and other related topics. Certainly, we all have a million reasons to miss his presence, friendship, and his unique laughter. As he used to say to compliment people, now we, on behalf of his peers and alumni, affectionately say to him: “*Rodrigo, you were a true spectacle*!”

## References

[1] Plataforma Lattes Rodrigo Correa Oliveira.

[2] Academia Brasileira de Ciências Rodrigo Correa Oliveira.

[3] Plataforma ORCID Rodrigo Correa Oliveira.

[4] Research.com Rodrigo Correa Oliveira.

